# One Health Paradigm to Confront Zoonotic Health Threats: A Pakistan Prospective

**DOI:** 10.3389/fmicb.2021.719334

**Published:** 2022-02-08

**Authors:** Nafeesa Yasmeen, Abdul Jabbar, Taif Shah, Liang-xing Fang, Bilal Aslam, Iqra Naseeb, Faiqa Shakeel, Hafiz Ishfaq Ahmad, Zulqarnain Baloch, Yahong Liu

**Affiliations:** ^1^National Risk Assessment Laboratory for Antimicrobial Resistance of Animal Original Bacteria, South China Agricultural University, Guangzhou, China; ^2^Guangdong Provincial Key Laboratory of Veterinary Pharmaceutics Development and Safety Evaluation, South China Agricultural University, Guangzhou, China; ^3^Faculty of Life Science and Technology, Kunming University of Science and Technology, Kunming, China; ^4^Department of Microbiology, Government College University, Faisalabad, Pakistan; ^5^Institute of Applied Microbiology, University of Veterinary and Animal Sciences, Punjab, Pakistan; ^6^Department of Animal Breeding and Genetics, University of Veterinary and Animal Sciences, Punjab, Pakistan

**Keywords:** zoonotic disease, One Health, human, livestock, Pakistan

## Abstract

The emergence and re-emergence of zoonotic diseases significantly impact human health, particularly those who live in impoverished areas and have close contact with domestic or wild animals. Nearly 75% of zoonotic diseases are transmitted directly from animals to humans or indirectly *via* vector/agent interactions between animals and humans. Growing populations, globalization, urbanization, and the interaction of the environment with humans and livestock all play roles in the emergence and spread of zoonotic diseases. “One Health” is a multidisciplinary concept aimed at improving human, animal, and environmental health, but this concept is not widely accepted in developing countries. In Pakistan, environmental, human, and animal health are severely affected due to a lack of sufficient resources. This review article provides an overview of the most common zoonotic diseases found in Pakistan and emphasizes the importance of the “One Health” concept in managing these diseases. Given the current situation, interdisciplinary research efforts are required to implement and sustain effective and long-term control measures in animal, human, and environmental health surveillance and accurate diagnostic methods.

## Introduction

**FIGURE 1:**
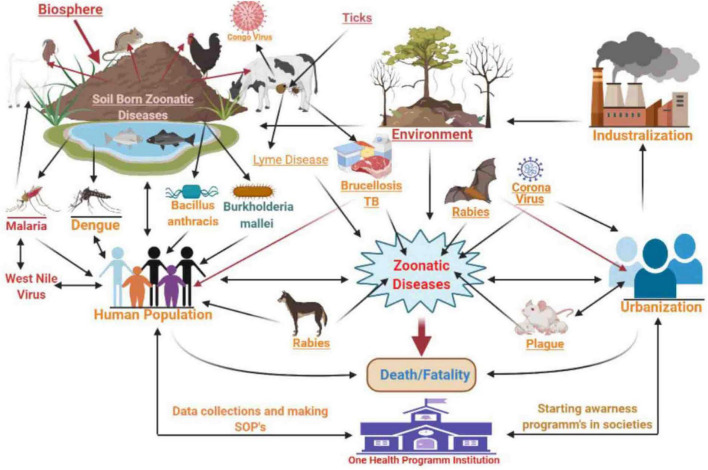
Zoonotic diseases and One Health concept.

Zoonotic diseases (zoonoses) are caused by microbes that are naturally transmitted from animals to humans. The ongoing occurrence of zoonoses pose significant threats to public health since nearly 60% of all infectious diseases are zoonotic and animal origins account for 75% of emerging transmissible infections (Mangili et al., 2016; Supramaniam et al., 2018; Espinosa et al., 2020). Zoonotic diseases are most commonly spread through direct contact from animals to humans or indirect contact (Figure 1) *via* vector/agent interactions (McArthur, 2019). Global environmental changes, increased populations, urbanization, animal migration, and tourism all play roles in the emergence of zoonotic diseases (Rahman et al., 2020). The “One Health” initiative that has been adopted by most industrialized countries allows for different sectors to collaborate in an effort to improve health outcomes. The goals are to promote and encourage a global health network by refining effective collaboration, cooperation, and contribution at the human-animal-environmental interface (McEwen and Collignon, 2018; Barton, 2019).

Pakistan is located near the Arabian Sea in South Asia and has the world’s sixth-largest population (208 million) (Pakistan Bureau of Statistics [PBS], 2017). Pakistan’s livestock population exceeds 300 M that includes 83 M large and 103 M small ruminants and 147 M poultry (Central Intelligence Agency [CIA], 2016). Regional conflicts in Afghanistan over the past 4 decades have resulted in massive refugee movements from Afghanistan to Pakistan (World Health Organization [WHO], 2004). Pakistan has a diverse natural topography, climate, and a wide range of domestic and wild animal species (Turnbull, 2008). Similarly, climate change, ecosystem diversity, poverty, social inequality, regional conflicts, and lack of a political will can all disrupt disease surveillance systems and public health (Ashraf et al., 2014).

The “One Health” concept has yet to be widely accepted in developing countries including Pakistan, where the prevalence of infectious diseases and hazardous biological materials has significantly negatively affected the environment and human and animal welfare. For example, a WHO report in 2017 documented >800 cases of Chikungunya virus infections in humans across Pakistan (World Health Organization [WHO], 2017). Similarly, Crimean–Congo Hemorrhagic Fever (CCHF) infected 63 people in Pakistan resulting in 11 fatalities (Altmann et al., 2019). Interactions between humans, animals, and the environment provide opportunities for pathogenic microbes to spread in any direction. Government institutions including the Ministries of Climate Change, Education, Industry and Food Safety as well as numerous non-governmental organizations (NGO) are responsible for designing and implementing innovative and practical strategies to control or prevent zoonotic diseases in Pakistan (Bartges et al., 2017). The current review provides an overview of the most common zoonotic diseases in Pakistan, and we focused on the importance of government and private sector collaborations to mitigate zoonotic threats according to the “One Health” concept.

### An Overview of Zoonotic Diseases in Pakistan

In Pakistan, common zoonotic diseases include tuberculosis (TB), rabies, encephalitis, Lyme disease, CCHF, foot and mouth disease, Brucellosis, Q fever, Leishmaniosis, Chagas disease/Trypanosomiasis, Balantidiasis, avian influenza, *Giardia*, and anthrax (Table 1; Feng and Xiao, 2011; Shabbir et al., 2015; Yousaf et al., 2018; Ahmed et al., 2020b; Iqbal et al., 2020a). Soil-borne zoonotic pathogens such as *Bacillus anthracis* and *Burkholderia mallei* have been reported in humans and animals in Punjab province (Shabbir et al., 2015). Moreover, DNA-based studies have revealed that *B. anthracis* has a high prevalence in Pakistan (Shabbir et al., 2015).

**TABLE 1:** List of common zoonotic diseases in Pakistan.

Zoonotic disease	Symptoms in human	Source of transmission	Risk factors	References
**Direct transmission/contamination**				
Salmonellosis	Fever, abdominal pain, diarrhea, vomiting, nausea	contaminated food, water, livestock products, contact with infected animals	Poor living conditions, lack of hygiene	Altaf Hussain et al., 2020; Petrin et al., 2020
Anthrax	Fever, headache, chills, nausea, sore throat, swelling of the neck, hoarseness, painful swallowing, vomiting, diarrhea	*Bacillus anthracis*: a soil-borne bacteria, transmitted *via* herbivores, spores, or *via* an infected carcass		Ahmad et al., 2004; Doganay and Demiraslan, 2015; Kim et al., 2015; Moayeri et al., 2015; Saad-Roy et al., 2017; Kolton et al., 2019
Food-borne *E. coli* infection	Fever, diarrhea, vomiting, respiratory disorders	Contaminated water, food, livestock products, contact with infected animals	Living conditions, lack of hygiene	Bokhari et al., 2013; Ishaq et al., 2021
Hepatitis E	Fever, yellow skin, tiredness, vomiting, nausea, abdominal pain, loss of appetite, liver failure	Food and water contaminated with human sewage, eating uncooked pig meat	Living conditions, lack of hygiene	Bosan et al., 2010; Butt and Sharif, 2016
Leptospirosis	Fever, headache, nausea, loss of appetite, jaundice, swollen limbs, chest pain, shortness of breath, coughing blood	contaminated soil and water with animal urine	Skin lesions/injuries, occupational exposure	Ijaz et al., 2018; Sohail et al., 2018
Bovine TB	Fever, weakness, loss of appetite, weight loss, intermittent cough, diarrhea, large prominent lymph nodes	Contaminated water, food, livestock products, unpasteurized dairy products, direct contact with infected animals	Animal husbandry; living conditions; occupational exposure; wildlife reservoirs	Awah Ndukum et al., 2010; Jafar et al., 2014
Brucellosis	Fever, weight loss, abdominal pain, weakness, body ache	Contact with aborted fetuses, vaginal fluids, placenta, milk, urine, semen, feces	Occupational exposure, ingesting unpasteurized dairy products	Bilal et al., 2009; Garshasbi et al., 2014; Mohammadi and Golchin, 2018; Qin et al., 2019
Rabies	Encephalitis, hyper-excitability, hydrophobia, motor neuron weakness, and paralysis	Animal bites (for example, dogs)	Free-roaming dogs, rarely pets	Ondrejková et al., 2015; Jackson, 2018; Singh and Ahmad, 2018; World Health Organization [WHO], 2018; Dascalu et al., 2019; Torquato et al., 2020
**Vector-borne diseases**				
Leishmaniosis	Fever, cutaneous leishmaniosis: skin lesions, weight loss, spleen, liver enlargement	Leishmania parasite transmission *via* female phlebotomine sandfly bite which feeds on blood; 70 animal species are natural reservoirs, including humans	Environmental changes, urbanization, malnutrition, people migration, unhygienic lifestyle, poor health status, poverty	Tiwananthagorn et al., 2012; Khan et al., 2016; Kämink et al., 2019
Chikungunya	Fever, joint pain/swelling, headache, muscle pain, skin rashes	This virus is maintained in the environment between humans, animals, and mosquitoes	Aedes mosquitoes transmit the chikungunya virus from infected to healthy people.	Ali and Dasti, 2018
Crimean-Congo hemorrhagic fever	Fever, myalgia, dizziness, neck pain/stiffness, headache, backache, nausea, vomiting, diarrhea, abdominal pain, sore throat, confusion, sleepiness, liver enlargement, petechial rash, liver failure	Tick bites, contact with infected livestock	Occupational exposure, human migration	Yousaf et al., 2018; Hatami et al., 2019; Kasi et al., 2020
Rift Valley Fever	Range from mild flu-like symptoms to severe hemorrhagic fever	Contact with infected livestock blood/organs, mosquito bites, unpasteurized milk	Occupational exposure	Atif et al., 2012
Foot and mouth disease	Fever, sore throat, pain, loss of appetite, red lesions on the tongue, gums, rashes on the palms, soles, buttocks, irritability in infants and toddlers	Cloven-hoofed animals, such as domestic and wild Bovidae, cattle, sheep, swine, humans	Small ruminants like sheep and goats can spread the virus	Ur-Rehman et al., 2014; Dubie and Amare, 2020

A significant proportion of human TB infections throughout the 19th and 20th centuries were caused by *Mycobacterium bovis* and raw cow milk consumption (Jafar et al., 2014). The large Pakistani livestock population is well-adapted to local environmental conditions and the source of 1.6 M tons of meat annually. Bovine TB infections are devastating for cattle (Jafar et al., 2014) and can transmitted to humans *via* aerosols in coughs, sneezes, or raw cow milk consumption. Pakistan ranks fifth among countries with a high TB burden (Shah et al., 2017) with 510,000 new TB cases emerging annually. For instance, a study of 3 primary abattoirs in Peshawar city that included lung and liver tissue samples from 121 buffaloes and 30 cattle indicated the presence of *M. bovis* in 4 cattle and 17 buffaloes with an overall prevalence of 10.18 and 11.53%, respectively (Jafar et al., 2014). This high prevalence of bovine TB was linked to a lack of preventive treatments, but the presence of the diseased animals in abattoirs indicates a lack of quality veterinary inspection and monitoring for the prevention and control of animal TB. Proper inspection and monitoring should be implemented to improve the quality of animal meat and to prevent TB transmission from diseased animals to humans.

Rabies virus belongs to the Rhabdoviridae family and is one of the deadliest single-stranded RNA viruses that infect both humans and warm-blooded mammals. These infections are most prevalent in bats, dogs, and raccoons, and these species spread the infection to humans. There are 5 million cases reported annually and 50,000 fatalities due to dog bites (World Health Organization [WHO], 2018). According to the National Rabies Control Program of Pakistan (NRCP), many rural areas in Pakistan are still at high risk of rabies and 54.7% of dogs that bit humans were not vaccinated against rabies (Noureen, 2018). In addition, another report indicated that 70 canine bite victims are treated in public and private hospitals daily. Therefore, the total incidence of rabies is likely in the range of 9 million (World Health Organization [WHO], 2018).

Multiple dengue outbreaks have been reported from different regions of Pakistan during the last three decades since the first outbreak was reported in 1994 (Khan and Khan, 2015; Abdullah et al., 2019; Junaidi, 2019; Fatima et al., 2021). In particular, the 2005 outbreak in Karachi involved >6000 cases and 52 fatalities In 2011, in Lahore there were >21,000 dengue cases and 350 fatalities (Junaidi, 2019), and in 2019, 44,415 people were affected and 66 died (Junaidi, 2019). Although the prevalence rate of the dengue virus has increased yearly, the overall mortality has decreased. This milestone has been achieved with the collaborative efforts of the WHO, local public and private institutions to promote screening, door-to-door surveillance, staff training, and to conduct organized awareness sessions with the public.

*Salmonella* Typhi causes typhoid fever and is commonly spread *via* contaminated water and food as well as through animal-to-human and person-to-person contact. Every year, 11–12 M typhoid cases are reported worldwide, and the estimated prevalence of typhoid fever is 451.7/100,000 (Ochiai et al., 2008). Therefore, there is a need for proper surveillance and monitoring strategies to control this disease (Fatima et al., 2021).

*Bacillus anthracis* causes anthrax and has a tremendous impact on animal health especially for cattle, sheep, and goats, and the infection is easily spread to humans. The likelihood of *B. anthracis* infections is greater in areas with many interactions between animals and humans such as slaughterhouses. An essential part of an effective disease surveillance program is animal vaccination and is needed for the prevention of future outbreaks.

Crimean–Congo Hemorrhagic Fever is one of Pakistan’s most lethal tick-borne viral diseases and is characterized by fever and hemorrhage. Rapid climate change has resulted in an increased prevalence of CCHF in Pakistan due to increased industrialization, agricultural and occupational activities, and population density. Factors that contribute to CCHF spread include poor sanitation in farms, villages, and cities, the unsanitary transportation and slaughter of animals within cities, ineffective tick-control programs, nomadic lifestyles, and a lack of trained healthcare staff. CCHF is present in most major cities including Karachi, Quetta, Peshawar, and Multan, and its transmission in Pakistan is linked to a lack of an effective disease surveillance system (Yousaf et al., 2018). The general public, farmers, and healthcare workers should be educated about CCHF transmission and its consequences by local and provincial governments. Implementation of a disease surveillance system, preventive measures, detection, and treatment are all urgently required to control and eradicate this lethal disease from the country.

### Potential Factors Necessary to Overcome Zoonotic Disease Prevalence in Pakistan

#### Surveillance of Disease Outbreaks

Zoonotic diseases are disseminated through animals, and disease outbreak surveillance helps to determine the cause, transmission, and pathogenesis and to guide prevention efforts (Supramaniam et al., 2018). Epidemiological surveillance is vital for population health management to determine associated risk factors responsible for disease persistence and spread. However, the collection of population-based data related to zoonotic disease prevalence in Pakistan is rare. Generally, hospital-based surveillance data has been used and indicates a high prevalence of numerous zoonotic diseases. Clinicians, epidemiologists, and specialists in environmental health and veterinary medicine can be brought together to formulate policies that address disease persistence. Awareness campaigns and vaccination programs that educate the general public should be instituted as well. Tools for programs such as these are included in the “One Health” system mapping and analysis resource toolkit (Breslin et al., 2017; Brown and Nading, 2019; Iqbal et al., 2020a). In addition, routine surveillance or the continuous declaration or reporting of diseases related to public health can also play an important role in preventing zoonotic diseases (George et al., 2020). Pakistan is an agricultural country, so it is also necessary to determine the prevalence and risks associated with new zoonotic pathogens in soil, plants, vegetables, and fruits and to have formulated an action plan for their prevention (Ahmed et al., 2017).

#### Environmental Change and the “One Health” Concept

Human expansion has increased atmospheric carbon emissions, resulting in an elevation of the global temperature that has disrupted normal lifecycles and ecosystems. Urbanization has accelerated the close contact of humans with animals such as squirrels, mice, jackals, foxes, and pigs. These are favorable circumstances for the emergence or re-emergence of zoonotic diseases (Sleeman et al., 2019). Deforestation and the loss of ecosystem diversity, air pollution from crop and coal combustion, melting ice due to global warming, extremes of temperature, over-population, increased humidity and temperatures, and decreased food production are having catastrophic effects on the natural diversity of the environment. These conditions also aid zoonotic pathogen survival and spread (El-Sayed and Kamel, 2020; Majeed and Munir, 2020). For instance, humidity and temperature directly influence the spread of the coronavirus through aerosols and the virus can be found in an active state for at least 2 weeks on most surfaces (Lin et al., 2020).

In Pakistan, inadequate infectious waste management is a major source of contamination in communities and facilitates infectious disease dissemination on a wide scale. Many hospitals and industries do not separate different types of waste materials such as chemicals, pulp, biologicals, textiles, and leather. These pollutants can act as carriers of hepatitis A and E, intestinal pathogens such as *Salmonella*, and acute respiratory disease pathogens (Rab et al., 1997; Qasim et al., 2014). The lack of proper industrial, hospital, farm, and household disposal can combine with natural events such as floods to contaminate drinking water supplies (Daud et al., 2017).

Enteric zoonotic diseases are often transmitted by the food chain and the environment (Klumb et al., 2020) such as bacteria residing in contaminated soil and surface waters (Vincent et al., 2019). Wildlife can also spread zoonotic pathogens to humans (Rothenburger et al., 2019). Disease burden is also tightly linked to poverty in Pakistan, and examples are dengue, *Vibrio cholera*, malaria, Lyme disease, COVID-19, influenza virus, respiratory syncytial virus, TB, and skin cancer (Baudouin et al., 2002; Patz et al., 2003; Khaliq et al., 2015; Velraj and Haghighat, 2020). Disease transmission due to the improper disposal of hazardous materials can be prevented, and this can begin at the community hospital level and successes can be used as examples for other areas including improvements for public trash disposal and limiting agricultural and industrial runoff (Ali, 2018; Hussain et al., 2020; Majeed and Munir, 2020). The “One Health” process has strategic plans that can be implemented to achieve these targets (Cunningham et al., 2017).

#### Animals and Food Safety

The prevalence of food-borne infections caused by *Listeria monocytogenes*, *Campylobacter* spp., *Salmonella* spp., *Toxoplasma gondii*, and Norovirus is common in Pakistan (Nisar et al., 2018). Furthermore, food is exposed to a variety of toxic chemicals during its preparation, processing, handling, and storage (Javed, 2016; Ishaq et al., 2021). Pakistan is one of the top milk and halal meat-producing states in the world, although the quality of these products is often not good. Therefore, diseases such as brucellosis and bovine TB begin at the farm level are transmitted during animal handling, milking, slaughtering, and processing (Claeys et al., 2013). The most successful method for improving milk production is pasteurization. Residual antibiotics as well as microbes and other contaminants in milk can be easily screened using Raman spectroscopic techniques (He et al., 2019). Pasteurization extends the shelf life of milk, and untreated or raw milk can contain infectious pathogens. Therefore, the latest milk production and processing techniques must be implemented on a country-wide basis to control milk-related zoonotic disease transmission in Pakistan.

Meat is a primary protein source and is consumed massively all over the world. The spread of food-borne illnesses such as Bovine Spongiform Encephalopathy (BSE), hepatitis, and typhoid has been linked to inappropriate meat processing (Ozawa, 2003; Javed, 2016), and standard operating procedures for farm and abattoir sanitation are necessary for healthy meat production. Environmental health practitioners play a crucial role in meat safety and the fulfillment of hygienic conditions. Food inspections are also required for meat labeled for export, and technologies like multiplex PCR for meat screening are relatively easy to implement (Iqbal et al., 2020b). All these safety procedures also apply to organic food production (Akbar et al., 2019). Safe meat for the consumer requires that quality standards are applied to animal handling, slaughtering, dressing, and storage (Ishaq et al., 2021).

#### Vectors and Their Controls

Climate change is a key player in the global spread of vector-borne diseases. According to WHO estimates, climate change will most likely cause 250,000 extra deaths annually due to malaria, malnutrition, heat stress, and diarrhea from 2030 to 2050. These climatic alterations result in heavy and unpredictable rainfalls, flooding, and high humidity, and all these are conducive for the propagation of disease vectors such as rodents, fleas, and mosquitoes (Ngeleja et al., 2017). Furthermore, air pollution from excessive gas emissions, the greenhouse effect, increased hydrocarbon combustion, and deforestation all contribute to a greater risk for zoonotic disease transmission (Rossati, 2017). Approximately half of the human population is at risk of vector-borne disease, and these diseases account for >17% of all infectious diseases and 1 M deaths worldwide each year. Increased temperature and humidity levels are linked to the surge in the prevalence of insect vector-borne diseases such as malaria, plaque, leishmaniosis, African trypanosomiasis, Japanese encephalitis, and diseases of viral origin like Rift Valley Fever. Additionally, health professional negligence has also been cited as a contributing factor to increased disease prevalence (Fouque and Reeder, 2019).

Pakistan is vulnerable to the impacts of climate change. The exact figure of vector-borne diseases in Pakistan is unclear, although there are currently 1.5 million malaria cases in Pakistan and these levels are comparable to Somalia, Afghanistan, and Djibouti. Inadequate housing, water, sanitation, and limited access to health facilities are the most direct possible causes of the prevalence of vector-borne diseases in Pakistan. The re-emergence of Leishmaniosis caused by the female phlebotomine sand fly (Khan et al., 2019), CCHF acquired *via* tick bites (Altmann et al., 2019), and Rift Valley Fever *via* mosquito bites or consumption of unpasteurized milk (Fouque and Reeder, 2019) are the major causes for morbidity and mortality levels that can all be addressed by the “One Health” Initiative. New strategies and collaborations with health administrations, the environmental ministry, entomologists, zoologists, veterinarians, and NGOs can assist vector-borne zoonotic disease management in Pakistan (Bostan et al., 2017; Huang et al., 2019).

#### Importance of Health Education

Health education is the building block of “One Health” where its motto is to educate the public about their health. Factors such as malnourishment, food insecurity, poverty, crowding, late reporting of disease, and poor observance of sanitary treatment measures as well as lack of vaccination and contaminated drinking water are major obstacles in securing Pakistan’s health security. In March 2002, 300 attorneys from 35 countries gathered to increase awareness and provide alertness related to environmental hazards and their effects on public health. Healthcare facilities should be updated in Pakistan, and awareness of these problems must be communicated through human resources to improve management systems particularly in rural areas (Ahmed and Shaikh, 2011). Information concerning food safety awareness and dietary guidelines to prevent zoonotic disease should be readily available to the general public. Further, policies for the improvement of farm production, sanitation, and food storage conditions should be implemented. Local authorities can develop collaborations with the Ministry of Health and Livestock for proper vaccination against zoonotic diseases, and overall, these measures will improve public health (Suk et al., 2003). This type of process would also assist in educating farmers and consumers in maintaining animal health. The focus on education is crucial because an understanding of a process is more likely to result in the acceptance of the values suggested by health services. But unfortunately, the Pakistani community is less likely to be aware of the basic issues due to a low literacy rate and a lack of understanding of how diseases spread and how these are connected with health parameters, socio-cultural and environmental problems, as well as political issues. Therefore, to overcome and halt this dangerous situation, it is necessary to adopt, enforce, and implement awareness campaigns especially related to health-seeking behavior and conduct in person surveys and develop assessment exercises with private and public sector collaborators (Shaikh and Hatcher, 2005; Zahid, 2018).

#### Population Density

The close association of humans and their livestock is linked to the transmission of zoonotic pathogens (Kilpatrick and Randolph, 2012; Suk et al., 2014), and these risks are elevated in areas where animals and humans share living areas (Nieto et al., 2012; Owczarczak-Garstecka, 2018) and those with inadequate sanitation facilities (Gayer et al., 2007; Warraich et al., 2011). When a disease has become established in a human population (Paterson et al., 2018; Braam et al., 2021), sedentary conditions in camps and informal settlements increase the risk of zoonotic pathogen transmission and population size and density affect a pathogen’s ability to infect susceptible hosts (Brooker et al., 2004; Hammer et al., 2018). Strengthening the standards for improving hygiene and sanitation in local food markets will decrease the risk of zoonotic disease transmission. In market settings, policies for crowd control, physical distancing measures, and handwashing and sanitizing stations should be implemented and enforced.

#### Poverty and Socio-Economic Inequities

Poverty and socioeconomic inequalities are associated with poor health (Vincent, 2016; Khan and Hussain, 2020). Disasters and displacement affect access to education, employment, and lifestyle choices and exacerbates poverty (Du et al., 2018). Displaced populations are frequently subjected to structural discrimination, violence, and a lack of equitable access to services (Castañeda et al., 2015). Furthermore, displaced communities are frequently located in geographically marginalized areas with limited resources (McMichael et al., 1998). For example, communities along the Pakistan-Afghan border bear the brunt of vector-borne disease due to human displacement (Nieto et al., 2012). Numerous events should encourage dialogue and collaboration with local representatives, academics, policymakers, and medical practitioners. Seminars and conferences should be held in various cities to raise public awareness about disease prevention, protect and strengthen investments in health and unemployment insurance, make tax systems less regressive, safeguard worker rights, and expand medical care facilities.

### Importance of Food and Water Safety and Its Hygiene Practices

Food is an important source of zoonotic disease. Zoonoses with a food-borne reservoir are typically caused by consuming food or contaminated water. Additionally, many zoonotic microbes exist in the gastrointestinal tracts of food-producing animals and poses a farm to fork risk of contamination. Therefore, food safety is a major concern for global public health (Gizaw, 2019) and pro-active strategies are required to mitigate the spread of these diseases (Chapman and Gunter, 2018; Ishaq et al., 2021). Food handling is a major factor in controlling the spread of food-borne diseases. It has always been challenging to control zoonotic diseases in countries (Ma et al., 2019), particularly Pakistan, where food is commonly sold in the streets under unsanitary conditions. Additionally, food safety knowledge in the general population is poor (Ma et al., 2019). These factors elevate the risk of zoonotic diseases such as the major pathogens *Salmonella*, *Campylobacter*, *Listeria*, *E. coli* O157:H7, *Bacillus cereus*, *and Clostridium* (Lammie and Hughes, 2016; Samad et al., 2018). The “One Health” concept promotes the wellbeing of humans and animals including farm and wild animals. This concept can decrease the prevalence of most food-borne diseases by using the combined efforts of environmental health professionals.

Water is the most fundamental resource, and pure drinking water is one of the most important components for life (Pandey, 2006). Unsafe or contaminated water can expose animals and humans to pathogens and pollutants resulting in gastrointestinal, neurological, and reproductive disorders (Lee and Murphy, 2020). Most of the water resources in the world have been polluted due to urbanization, industrialization, and environmental changes (Pandey, 2006). Therefore, approximately 2.2 billion people are using unsafe drinking water in the world (World Health Organization [WHO], 2019).

Pakistan is a developing country located in South Asia. Urbanization, industrialization, and population growth have polluted water resources in Pakistan. Therefore, only 20% of the population has access to safe drinking water in Pakistan (Daud et al., 2017). In terms of potable water quality, Pakistan has been ranked 80th out of 122 countries. If water quality issues are not addressed, then a 60% potable water shortage may soon be confronted due to the mixing of community, sewerage, and industrial waste without treatment (Aziz, 2005; Azizullah et al., 2011; Bhowmik et al., 2015; Khalid et al., 2018; Ilyas et al., 2019). In 2020, 400+ schools were randomly tested in Pakistan to examine the quality of the water and >50% of the samples were contaminated with highly pathogenic microorganisms (Ahmed et al., 2020a). Another study reported that drinking water in Sibi district, Baluchistan was highly contaminated with fluoride and arsenic (Chandio et al., 2020). Contaminated or untreated water originating from agriculture is also a major issue in Pakistan (Shahid et al., 2020), and crops produced from contaminated water are not fit for human consumption. In Pakistan, water supplies have been adversely influenced by climate change, chemical and biological pollutants due to pipe cracks, poor sewage systems, and a lack of water quality control testing systems (Aziz, 2005). Governments and NGOs have adopted different rules and regulations to control the risk of water contamination such as the WASH interventions, Water and Sanitation Extension Programs (WASEP) projects, Oxfam GB, Quantitative Microbiological Risk Assessment (QMRA), Punjab Saaf Pani project, and the Changa Pani scheme (Nanan et al., 2003; Baig et al., 2012; Ahmed et al., 2020a,c; Als et al., 2020). WASEP projects are particularly enforced at the rural level to enhance or rectify water supplies for consumers (Nanan et al., 2003). Similarly, the NGO Oxfam GB is putting its efforts into evaluating major problems associated with the quality of potable water (Baig et al., 2012). In addition, the Punjab Saaf Pani and Changa Pani projects are contributing to the improvement of water quality in rural and urban areas (Ahmed et al., 2020c).

Organizations such as the Pakistan Environmental Protection Council (PEPC) and the Pakistan Environmental Protection Agency (Pak-EPA) are contributing their efforts to implementing protective standards such as the National Environmental Quality Standard (NEQS). The primary concern is how to perform technical assessments of water quality and provide microbiologically certified safe water by following the recommended procedures of the Environment, Health and Safety (EHS) ministry. Unfortunately, these organizations were unable to apply those environmental safety standards to all industrial and non-industrial sectors (Azizullah et al., 2011). This situation can be remedied by implementing suitable holistic solutions and legislation through proper monitoring systems in all national, local, and individual sectors.

## Conclusion

Zoonotic diseases pose the largest challenge for developing countries because humans, animals, and the environment all play roles in their transmission. Pakistan faces huge challenges due to a lack of strategic planning for responses to zoonotic disease infections. The “One Health” strategy can assist governmental agencies such as the Ministries of Climate Change, Education, Industry and Production and Food Safety by collaborating with the private sector and NGOs to adopt innovative and practical plans to control or prevent zoonotic diseases in Pakistan. The environmental health and food supply chains require a “One Health” approach to deal with zoonotic diseases (Lammie and Hughes, 2016).

## Author Contributions

YL designed the study. NY, AJ, and ZB wrote the initial version of the manuscript. ZB, TS, L-XF, and BA revised the final draft of the manuscript. IN, FS, and HA searched the literature and designed the table and figure.

## Conflict of Interest

The authors declare that the research was conducted in the absence of any commercial or financial relationships that could be construed as a potential conflict of interest.

## Publisher’s Note

All claims expressed in this article are solely those of the authors and do not necessarily represent those of their affiliated organizations, or those of the publisher, the editors and the reviewers. Any product that may be evaluated in this article, or claim that may be made by its manufacturer, is not guaranteed or endorsed by the publisher.
